# Effect and Mechanism Analysis of Process Parameters and Penetration State on Pore Defects of 1060/2A12 Dissimilar Aluminum Alloy Electron Beam Welding Joints

**DOI:** 10.3390/ma18153477

**Published:** 2025-07-24

**Authors:** Guolong Ma, Gangqing Li, Xiaohui Han, Chenghui Jiang, Zengci Cheng, Wangzhan Diao, Houqin Wang

**Affiliations:** 1 Technical Engineering Department, CRRC Qingdao Sifang Co., Ltd., Qingdao 266111, China; 2 State Key Laboratory of Precision Welding & Joining of Materials and Structures, Harbin Institute of Technology, Harbin 150001, China; 3 Kunming Institute of Physics, Kunming 650223, China; 4 State Key Laboratory of Low-Carbon Thermal Power Generation Technology and Equipments, Harbin 150001, China; diaowz@hbc.com.cn; 5 Harbin Boiler Company Limited, Harbin 150036, China

**Keywords:** electron beam welding, penetration state, cavitation potential, keyhole stability, pore defects

## Abstract

Pore defects are one of the most common defects in the aluminum alloy electron beam welding process. In this paper, research on the pore defects and related mechanisms of the electron beam welding of dissimilar aluminum alloys was carried out with 1060 and 2A12 aluminum alloys. Under the test conditions, the pore defects of the aluminum alloy joint were related to the penetration status, the porosity of the critically penetrated joint was low, and the porosity of the beam joint increased when it was slightly penetrated. When the welding speed changed from 300 mm/min to 1200 mm/min, the porosity in the critically penetrated joint first increased and then decreased. When the welding speed was set to 300 mm/min and the beam current was set to 26 mA, the porosity of the joints reached its minimum value at 0.23%. Based on the actual process of electron beam welding, a flow simulation model was established to study the aluminum alloy welding process. The stability of the keyhole was related to the electron beam energy density reaching the inner keyhole, so increasing the electron beam for the fully penetrated joints was advantageous for reducing the pore defects.

## 1. Introduction

Al 1060, classified as an industrial pure aluminum, has found extensive applications across various industrial sectors due to its advantageous properties, including low density and excellent weldability [1]. Aluminum alloy has the advantages of high specific strength and stiffness, high thermal conductivity, and strong corrosion resistance, which makes it an important light metal material widely used in the aviation and aerospace industries [2,3]. Electron beam welding is a high-energy-density welding method that can obtain welded joints with a large aspect ratio, small welding deformation, and heat-affected zone. It has significant advantages in aluminum alloy welding [4,5]. Due to aluminum alloy’s low melting point, fast heat conduction, and more low-melting volatile components, the gas in the molten pool is too slow to escape and form pore defects under rapid cooling conditions, which will affect the service performance of the aluminum alloy [6,7,8]. These adverse effects even limit its application in industry. Therefore, it is imperative to find an effective method for selecting the appropriate welding process parameters.

Much research has been performed on pore formation in aluminum alloy welding. In laser welding and electron beam welding joints of aluminum alloys, the pore defects are divided into metallurgical pores and process pores [9]. Metallurgical pores have a regular shape and smooth inner wall [10]. Process pores exhibit irregular morphologies characterized by step-like features on internal surfaces [11]. Metallurgical pores in aluminum alloy welds are primarily attributed to the vaporization of low-boiling-point elements (e.g., Mg) or hydrogen desorption within the molten pool [12,13], while process-induced pores are commonly associated with keyhole instability-induced cavity formation during keyhole collapse. The effects of the energy input modes and beam scanning strategies on weld porosity in aluminum alloys have been systematically investigated [10]. Conduction mode welding demonstrates superior porosity mitigation compared to the keyhole mode, attributed to enhanced gas escape channels and prolonged solidification durations. Beam oscillation further reduces defects through enhanced molten pool convection [14,15,16,17,18]. While substantial evidence exists regarding the penetration state effects on porosity, the mechanistic correlation between penetration characteristics and porosity formation remains unexplored.

Scholars have analyzed the gas distribution and bubble flow using the method of welding molten pool flow simulation, and have further clarified the mechanism of pore generation. Yi [19] established a molten pool bubble flow model for vacuum electron beam welding of magnesium alloys to analyze the formation and distribution of pore defects. It was found that the fluid flow rate in the center of the unmelted molten pool was faster, and the gas tended to accumulate here, which was not conducive to escape. The gas in the molten pool that has penetrated relatively can escape from the upper and lower surfaces of the molten pool at the same time. The higher liquid convection velocity was more conducive to the escape of gas in the molten pool. Chinnapat P’s study [20] showed that the wall of the keyhole had irregular undulations and trapped the pores, thereby inducing the generation of pores. The three-dimensional model of laser deep penetration welding established by Pang [21] showed that the center of the keyhole produces a large metal vapor reaction force, which encourages the liquid to flow outside the molten pool. This form was not conducive to the floating of bubbles and the escape of gas, resulting in an increase in the number of pores. The results of Komkamol [22] showed that the temperature in the keyhole area was high and the surrounding temperature was low. When the keyhole was formed, the density of the surrounding material increased and the volume of the molten pool expanded. The bubbles at the bottom of the keyhole form pores after the weld is cooled. Elucidating the formation mechanisms of porosity in aluminum alloy welding through the simulation of molten pool dynamics and keyhole stability represents a scientifically robust methodology for analyzing defect generation processes.

In this paper, electron beam welding of 1060/2A12 dissimilar aluminum alloys was performed and the effects of different beam currents and welding speeds on the number and porosity of welded joints is compared. Subsequently, a numerical simulation finite element model is established to analyze the formation mechanism of pores in welded joints under different process parameters and penetration states. The optimal welding process parameters for minimizing porosity defects are given by combining the results of the experiments and numerical simulation.

## 2. Experimental Procedure

**Figure 1:**
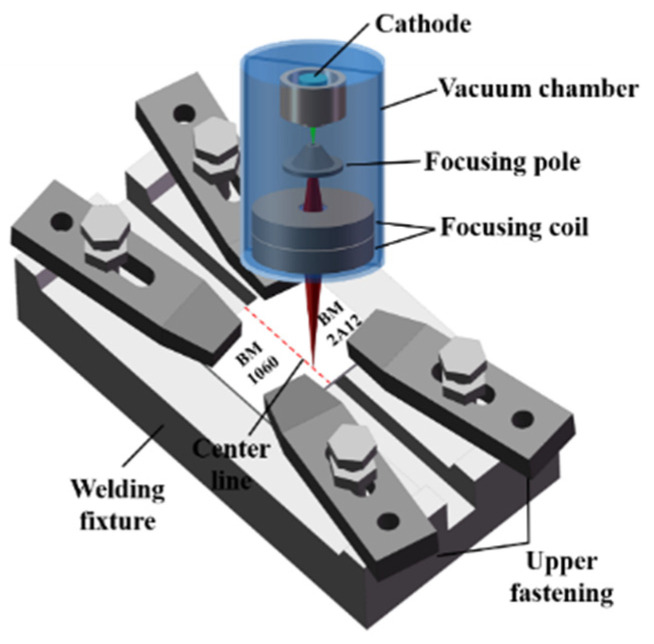
Schematic diagram of welding.

The test materials were 1060 and 2A12 aluminum alloys, and their chemical compositions are shown in Table 1. They were cut into a size of 50 mm × 100 mm × 5 mm. The samples were fixed with a special fixture to ensure the surfaces could be contacted closely and the gap between the plates was small, as shown in Figure 1. The equipment used in the experiment was the HIT-951 welder with a maximum accelerating voltage of 60 kV). Before welding, the two aluminum alloy surfaces were cleaned. The steps were as follows: sandpaper grinding, 10 NaOH solution corrosion, 1:5 nitric acid solution neutralization, mechanical cleaning, alcohol wipe, and drying. The sample to be welded was put into the vacuum chamber and the vacuum degree was pumped to a high vacuum state of 5 × 10^−4^ torr before welding. The welding parameters of the two dissimilar aluminum alloys are shown in Table 2. In all experiments, the process parameters that needed to be juxtaposed were beam current I_b_ and welding speed v, while the others remained unchanged. Accelerating voltage U was 60 kV; focusing current was 486 Ma; working distance was 253 mm.

The XT2005D X-ray flaw detector produced by Xinke Electrical Appliance Co. (Guangzhou, China), LTD was used to detect the number and distribution of pores in the welded joint after welding. The images were processed using LabVIEW 2020 software for binarization to quantify the projected area of pores along identical lengths. The porosity (defined as the percentage of pore-projected area relative to the total weld seam-projected area) and pore quantity were statistically analyzed. After non-destructive testing, the longitudinal section and cross-section of the stable section of the welds were cut to take out samples of different sizes, and then sandpaper with grit sizes from 400 to 7000 was used to polish until the surface had no obvious coarse scratches. Firstly, the specimens were mechanically polished by using a diamond polishing agent with a particle size of 2.5 μm, hereafter changing to a polishing agent with a particle size of 1 μm for fine polishing until the specimens’ surfaces were bright. In order to reveal the microstructure, the polished specimens were corroded for 10 s, for which the Keller reagent (1%HF + 1.5%HCl + 2.5%HNO_3_ + H_2_O) was applied as an etchant. A VHX-1000 ultra-depth optical microscope was used to observe the shape and distribution of the pores in the welding joints.

## 3. Numerical Simulation Modeling

### 3.1. Modeling Assumptions and Governing Equations

#### 3.1.1. Modeling Assumptions

**Figure 2:**
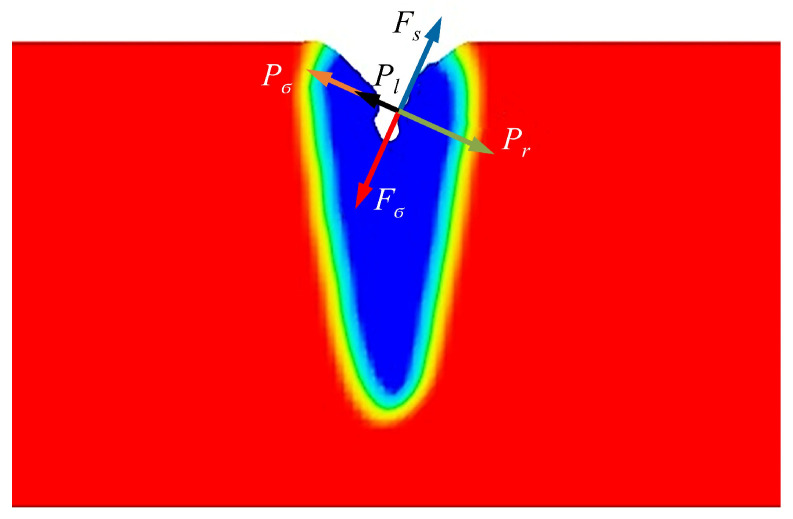
Schematic diagram of keyhole force.

In this research, the CFD commercial software Flow 3D 11.2, which can add required subroutines written in FORTRAN, is used to simulate and compute the welding process. The factors that affect the formation of the keyhole in the electron beam welding process are very complicated, and the force diagram of the keyhole wall is shown in Figure 2. EBW involves an involuted multi-field coupling phenomenon including complex multiphase transformation, free surface keyhole evolutions, heat transfer, and fluid flow in the molten pool. So, to improve the accuracy and efficiency of numerical calculations, it is necessary to simplify the model and make some reasonable assumptions. The following assumptions and simplifications have been applied in this paper [25]:

(1) Only the metal vapor reaction force $\vec{F_{r}}$, surface tension $\vec{F_{\sigma }}$, and gravity G are considered as three main forces that affect the flow of liquid metal in the molten pool.
(2) The EBW process includes the initial stage, quasi-steady-state stage, and end stage. The model only considers the flow state of the molten pool in the quasi-steady-state stage.
(3) The initial working temperature is 293 K, and the air pressure in the vacuum area is set to 0 Pa.
(4) The liquid metal inside the molten pool is a Newtonian fluid, which does not consider the flow of metal vapor and plasma inside the keyhole.
(5) The specific heat capacity, dynamic viscosity, and thermal conductivity are all functions of temperature, and other metal properties are set as constants. The material is isotropic.

#### 3.1.2. Governing Equations

The flow of aluminum alloy molten pool is governed by the law of physical conservation. The fluid movement must satisfy the basic law of conservation. Three conservation equations of CFD (computational fluid dynamics) to describe the molten pool flow are as follows:

(1) Mass conservation equation:

(1) $$\frac{\partial \rho }{\partial t}+\Delta \cdot (\rho \vec{V})=0$$

In this formula, $\Delta \cdot (\rho \vec{V})=\partial (\rho u)/\partial x+\partial (\rho v)/\partial y+\partial (\rho w)/\partial z$ in which *ρ* is density; t is time; $\vec{V}$ is fluid velocity; u, v, and w represent the velocity components in the x-, y-, and z-directions, respectively.

(2) Momentum conservation equation (Navier–Stokes equation):

x-direction:

(2) $$\begin{array}{l} \frac{\partial (\rho u)}{\partial t}+\frac{\partial (\rho uu)}{\partial x}+\frac{\partial (\rho uv)}{\partial y}+\frac{\partial (\rho uw)}{\partial z}=\\ -\frac{\partial P}{\partial x}+\frac{\partial }{\partial x}(\mu \frac{\partial u}{\partial x})+\frac{\partial }{\partial y}(\mu \frac{\partial u}{\partial y})+\frac{\partial }{\partial z}(\mu \frac{\partial u}{\partial z})+S_{u} \end{array}$$

y-direction:

(3) $$\begin{array}{l} \frac{\partial (\rho v)}{\partial t}+\frac{\partial (\rho vu)}{\partial x}+\frac{\partial (\rho vv)}{\partial y}+\frac{\partial (\rho vw)}{\partial z}=\\ -\frac{\partial P}{\partial y}+\frac{\partial }{\partial x}(\mu \frac{\partial v}{\partial x})+\frac{\partial }{\partial y}(\mu \frac{\partial v}{\partial y})+\frac{\partial }{\partial z}(\mu \frac{\partial v}{\partial z})+S_{v} \end{array}$$

z-direction:

(4) $$\begin{array}{l} \frac{\partial (\rho v)}{\partial t}+\frac{\partial (\rho vu)}{\partial x}+\frac{\partial (\rho vv)}{\partial y}+\frac{\partial (\rho vw)}{\partial z}=\\ -\frac{\partial P}{\partial y}+\frac{\partial }{\partial x}(\mu \frac{\partial v}{\partial x})+\frac{\partial }{\partial y}(\mu \frac{\partial v}{\partial y})+\frac{\partial }{\partial z}(\mu \frac{\partial v}{\partial z})+S_{v} \end{array}$$

where p is fluid microelement pressure; μ is viscosity coefficient; S_u_, S_v_, and S_w_ are source terms of generalized momentum equation in x-, y-, and z-directions, respectively. S_u_ = F_x_ + S_x_, S_v_ = F_y_ + S_y_, S_w_ = F_z_ + S_z_, and for incompressible Newton fluids, S_x_ = S_y_ = S_z_ = 0.

Gravity G is included in the momentum source term, with F_x_ = F_y_ = 0, F_z_ = G_z_ = −9.81 m/s^2^.

(3) Energy equation:

(5) $$\frac{\partial (\rho H)}{\partial t}+\nabla \cdot (E\vec{V})=\nabla \cdot (k\nabla T)+q_{sor}$$

where E is internal energy of materials; k is thermal conductivity; q_sor_ is energy source term; and the viscous heat of liquid metal is not considered, so q_sor_ = 0.

The internal energy E of the material corresponding to the temperature T_0_ can be calculated using the following equation:

(6) $$E(T_{0})=\rho (T_{0})\cdot [\Delta H_{m}+\int_{0}^{T_{0}}C_{p}(T)dT]$$

where ΔH_m_ is melting enthalpy; C_p_ is capacity.

(4) Liquid–gas interface tracking equation:

During the electron beam welding process, the material melts under the action of the electron beam heat source. When the surface temperature of the molten pool reaches the vaporization temperature of the base material, the violent vaporization of the materials exerts downward pressure on the molten pool, which leads to the formation of keyholes and serious topological deformation of the gas–liquid interface. In order to effectively track the gas–liquid interface of the small hole, the VOF method based on the Euler grid system is selected. This method defines the liquid volume fraction F in the discrete grid, and obtains the free surface by solving the change in the F value of each grid in the iterative process. F(x,y,z,t) = 1 means that the grid is completely filled with liquid, while F(x,y,z,t) = 0 means that there is no liquid in the grid. When the value of F on the grid where the liquid–gas interface is located satisfies 0 < F(x,y,z,t) < 1, the governing equation of fluid volume fraction F is

(7) $$\frac{\partial F}{\partial t}+\frac{\partial }{\partial x}(F\Delta H_{x}u)+\frac{\partial }{\partial y}(F\Delta H_{y}v)+\frac{\partial }{\partial z}(F\Delta H_{z}w)=0$$

where Δ*H_x_*, Δ*H_y_*, and Δ*H_z_* are melting enthalpy; u, v, and w are the fluid velocity in the x-, y-, and z-directions, respectively.

### 3.2. Mesh Generation and Boundary Conditions

#### 3.2.1. Mesh Generation

**Figure 3:**
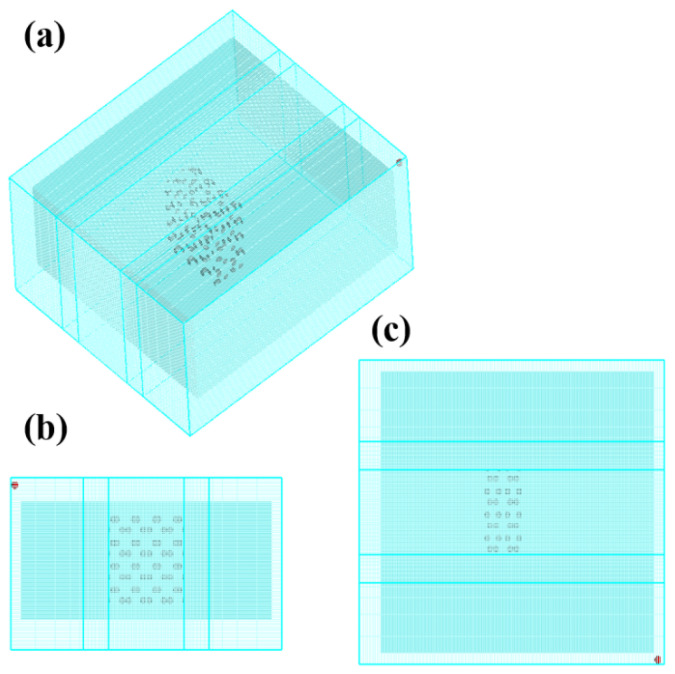
Geometric model and meshing generation: (**a**) basic model, (**b**) front view, and (**c**) top view.

The geometric model and mesh division of the molten pool flow field are shown in Figure 3. The actual size of the welded butt sample is 100 mm × 100 mm × 5 mm. To ensure the calculation speed and accuracy when building the model, the applied model size is 13 mm × 14 mm × 5 mm. The grid size in the thickness direction (z-direction) is set to 0.080 mm. Non-uniform grids are used along the x- and y-directions and dense grids are used in the area of −1.8 mm ≤ x/y ≤ 1.8 mm, of which the size is 0.080 mm. The maximum grid size in the x-/y-direction is 1.2 mm, and they are used as transition grids when −3.0 mm ≤ x/y ≤ −1.8 mm and 1.8 mm ≤ x/y ≤ 3.0 mm, respectively. The mathematical model contains a total of 1,214,668 calculation units. The preliminary experiments have verified that local mesh refinement does not compromise the accuracy of the simulation results.

#### 3.2.2. Boundary Conditions

To solve the governing equations above, setting corresponding boundary conditions is needed, which can realize the simulation process of molten pool flow in EBW of aluminum alloy. The boundary conditions are as close as possible to the actual welding process and are shown as follows:

(1) Initial conditions

The temperature of the specimen is set as room temperature before welding, and the velocity of the weld is 0. The initial conditions are as follows:

(8) $$T=T_{ref}$$

(9) u=v=w=0

(2) Momentum boundary conditions

The surface of the molten metal is subjected to surface tension ${\vec{F}}_{\sigma }$ and metal vapor reaction force ${\vec{F}}_{r}$, which are momentum boundary conditions applied to the keyhole wall.

Surface tension acts on the keyhole wall to promote the closure of the keyhole during the welding process, the additional pressure of which is P_σ_ at the bending interface of the molten pool. Moreover, the surface tension of liquid aluminum decreases with increasing temperature , and the change in the surface tension caused by the temperature gradient causes a tangential force f_l_ on the surface of the molten pool. The combined force of the above two is calculated as follows [26]:

(10) $${\vec{F}}_{\sigma }=\sigma k\hat{n}+\nabla \sigma =\sigma k\hat{n}+\frac{\partial \sigma }{\partial T}\frac{dT}{dl}$$

(11) $$\sigma (T)=\sigma_{m}-\gamma (T-T_{m})$$

where σ is the surface tension coefficient; σm is the surface tension of the material at the melting point Tm; γ is the gradient of the temperature coefficient of the surface tension, the values of which for 1060 and 2A12 are 1.55 × 10^−4^ and 3.5 × 10^−4^ respectively; k is the surface curvature of the molten pool; and $\hat{n}$ is the normal unit vector of the free surface of the molten pool.

The metal vapor reaction force promotes the formation of keyholes in the electron beam welding process. When the metal temperature reaches the boiling point of the material, it evaporates strongly with recoil pressure on the liquid pool, which increases the penetration. Its equation is [27]

(12) $$F_{r}\approx 0.21P_{0}\exp[\frac{M\cdot \Delta H_{vap}}{R}(\frac{1}{T_{0}}-\frac{1}{T_{l}})]$$

(3) Energy boundary conditions

Liquid metal will absorb heat when it is converted into gas, so the vaporization heat loss caused by metal evaporation q_ev_ should be considered in the simulation:

(13) $$q_{ev}=m\cdot \Delta H_{vap}$$

### 3.3. Heat Source Model and Material Thermo-Physical Parameters

In this research, a rotary Gaussian surface heat source was employed to describe the energy distribution of the electron beam, which can be mathematically expressed as follows:

(14) $$q(x,y,z)=\frac{3\eta Q}{\pi r_{H}^{2}}\exp(\frac{-3r^{2}}{r_{H}^{2}})$$

where q is the electron beam power density; Q is the electron beam power; η is the electron beam heating efficiency; r_H_ is the characteristic radius of the electron beam heat source; and r is the radial distance from any point on the surface of the molten pool to the center of the electron beam.

**Figure 4:**
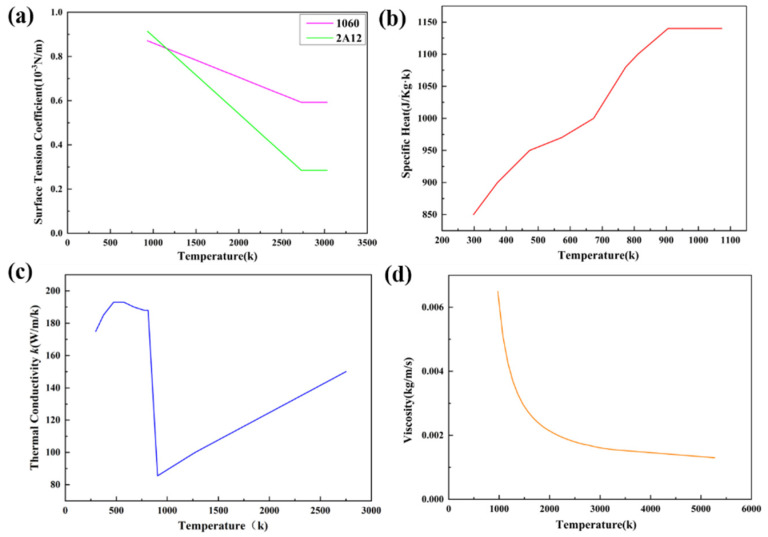
Thermo-physical parameters of base metals: (**a**) surface tension coefficient, (**b**) specific heat, (**c**) thermal conductivity, and (**d**) viscosity.

The materials used for model setting were 1060 industrial pure aluminum and 2A12 aluminum alloy. It is necessary to pay attention to the changes in the thermal conductivity, specific heat, surface tension, and viscosity coefficient with temperature during simulation calculations. Low temperature parameters can be obtained by consulting the data, while for high temperature parameters, which were difficult to obtain by consulting data, these can be obtained by extension or fitting. The solidus and liquidus temperature of 1060 aluminum is 933 K, and for 2A12 aluminum alloy, which contains alloying elements such as Cu and Mg, its solidus and liquidus temperatures are 811.15 K and 905.15 K, respectively. Figure 4 is the schematic diagram of the thermo-physical parameters changes in the two materials with temperature. It was found that their surface tensions are quite different.

Surface tension of 1060 (15) and 2A12 (16):

(15) $$\sigma =0.871-1.55\times 10^{-4}\cdot (T-933.35)$$

(16) $$\sigma =0.914-3.5\times 10^{-4}\cdot (T-930)$$

## 4. Results and Discussion

**Figure 5:**
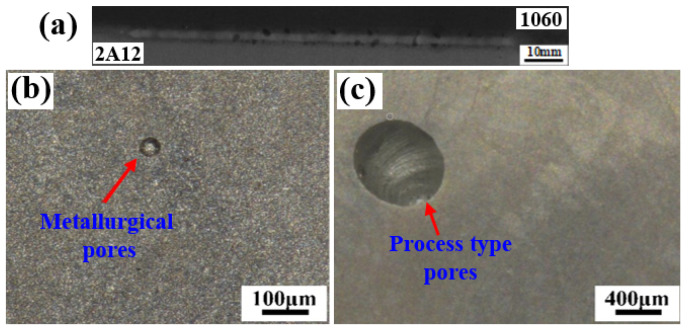
Pore defects of 1060/2A12 dissimilar aluminum alloy joints: (**a**) X-ray diffraction image; (**b**) metallurgical pores and (**c**) process-type pores.

Figure 5a shows the results of X-ray non-destructive testing of 1060/2A12 dissimilar aluminum alloy joints obtained by electron beam welding. In the picture, the gray base material is 1060 aluminum, and the lighter base material is 2A12 aluminum alloy. It was found that there were serious pore defects in the welding joints of the dissimilar aluminum alloys of 1060 and 2A12. According to the morphological characteristics of the pores, the pore defects in the weld were divided into metallurgical pores and process-type pores, as shown in Figure 5b,c.

### 4.1. Influence of Process Parameters

#### 4.1.1. Beam Current

**Figure 6:**
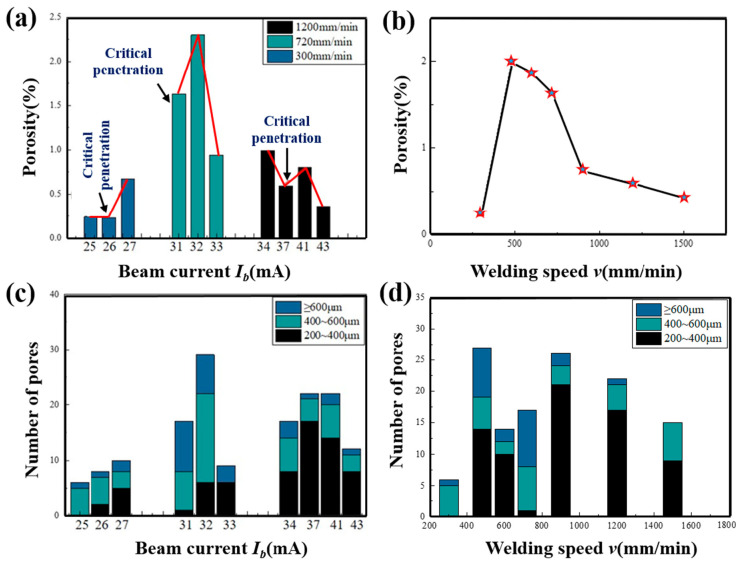
Porosity and number of pores under different process parameters: (**a**,**c**) beam current, (**b**,**d**) welding speed.

When welding aluminum alloy, heat input is one of the main factors affecting the stability of the keyhole and the flow behavior of the molten pool [28]. In this paper, the influence of beam current on pore defects of dissimilar aluminum alloy welds is studied under three speed levels. The X-ray inspection image was binarized with Labview 2020 software and the projected area of the pores on the same weld length was counted to calculate the porosity of the joints (projected area’s percentage of pores in the area of the weld), shown in Figure 6a. The joint porosity obtained at the two welding speeds of 300 mm/min and 1200 mm/min was maintained at a relatively low level. When the speed was 300 mm/min and the beam current was 26 mA, the porosity was 0.23%, and it was 0.36% at a speed of 1200 mm/min and beam current of 43 mA. When the welding speed was 720 mm/min, the overall porosity of the joint was higher. Further analysis showed that the porosity of the joints obtained at different speeds reached a lower level at the critical penetration state. Then, the porosity of the welds was maximized by adding one beam current to the micro-penetration state of the beam joint, and the porosity of the joint will decreased with the increase in the beam current. Figure 6c shows the result from counting the number of different sizes of pores by X-ray. At a speed of 300 mm/min, the number of pores was the least, while at a speed of 720 mm/min, there were lots of pores of a size over 600 μm in the joints. For the aluminum alloy joint obtained at the speed of 1200 mm/min, the number of pores with a size between 200 and 400 μm was the largest.

#### 4.1.2. Welding Speed

The welding speed can affect the solidification rate of the molten pool metal. When the bubble escape rate is less than the solidification rate of the liquid metal, the gas trapped in the molten pool metal after solidification will form pore defects. Moreover, the welding speed is also one of the main factors affecting the flow behavior of the molten pool, and the flow behavior has a greater impact on the bubble escape process. Since the porosity of the weld is greatly affected by the penetration state of the joint, this paper investigates the influence of the welding speed on the joint porosity under the condition of ensuring the critical penetration of the joint.

Figure 6b showed the porosity of dissimilar aluminum alloy joints at different speeds. As the welding speed increases from 300 mm/min to 1500 mm/min, the joint porosity and the number of pores both increase first and then decrease. When the speed was 480 mm/min, 600 mm/min, and 720 mm/min, the joint porosity for all was above 1.5%. There were more large-sized pores in the critical penetration welds obtained under these three beam levels, as seen in Figure 6d.

To observe the shape and distribution of the pores, the joints welded at different speeds were cut along the longitudinal section. It can be seen from Table 3 that there were no irregularly shaped process-characteristic pores on this section at the speed of 300 mm/min. When the speed was 720 mm/min, there were a lot of pore defects distributed on the section, and the metallurgical pores were mostly distributed in the lower part, while the process-type pores were mostly distributed in the upper part of the section. In addition, with the increase in the welding speed, the position of the pores gradually moved down. The analysis suggests that this may be due to the short existence of the molten pool when the welding speed is fast, and the bubbles do not have enough time to rise.

### 4.2. Analysis of Mechanism

Through the process test and simulation test, it was found that it took a long time to reach the steady state in the electron beam welding of the aluminum alloys. Considering the possibility of creating a model, in this section, we carry out a simulation calculation for the electron beam welding of the 2A12 aluminum alloy with a thickness of 2.5 mm.

#### 4.2.1. Keyhole Behavior

**Figure 7:**
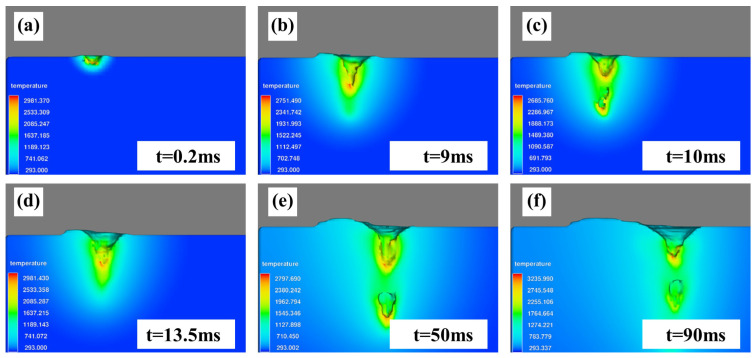
Keyhole evolution process: (**a**) 0.2 ms, (**b**) 9 ms, (**c**) 10 ms, (**d**) 13.5 ms, (**e**) 50 ms, and (**f**) 90 ms.

The keyhole formation process in electron beam welding is shown in Figure 7. When the surface is just heated by the electron beam heat source, the molten pool temperature is not high, and the metal vapor reaction force is small, which can make the molten pool surface concave under its action. When 0.2 ms ≤ t ≤ 0.9 ms, the molten pool temperature will keep rising due to heat accumulation during the heating process, with metal evaporation enhancing this. Under the strong action of the metal vapor reaction force, molten pool liquid is discharged to form a keyhole. At the same time, the bottom liquid of the keyhole is squeezed and flows to the back of the molten pool. The keyhole temperature is approximately in accordance with the Gaussian distribution, in which the bottom temperature of the keyhole is the highest, and the smaller the depth of the keyhole, the lower the temperature. Therefore, the metal vapor reaction force on the upper keyhole is so small that the liquid here fills the keyhole under the action of surface tension, and the molten pool surges forward. Due to the loss of vaporization heat caused by metal evaporation, some of heat dissipates because of the overheated liquid flowing to the back of the molten pool, and the electron beam heat source is prevented from acting on the bottom of the keyhole by a hump formed on the keyhole wall. Then, when the keyhole fluctuates, the bottom temperature of the keyhole reduces, and the metal vapor reaction force is weakened, such that the keyhole closes under the action of surface tension. When the keyhole closes, the surface temperature of the molten pool rises under the heat source, and the metal vapor reaction force is strengthened correspondingly, leading to the formation of the keyhole again. Repeatedly, the depth of the molten pool continues deepening to a steady state [29,30].

#### 4.2.2. The Influence Mechanism of Penetration State on Metallurgical Pores

**Figure 8:**
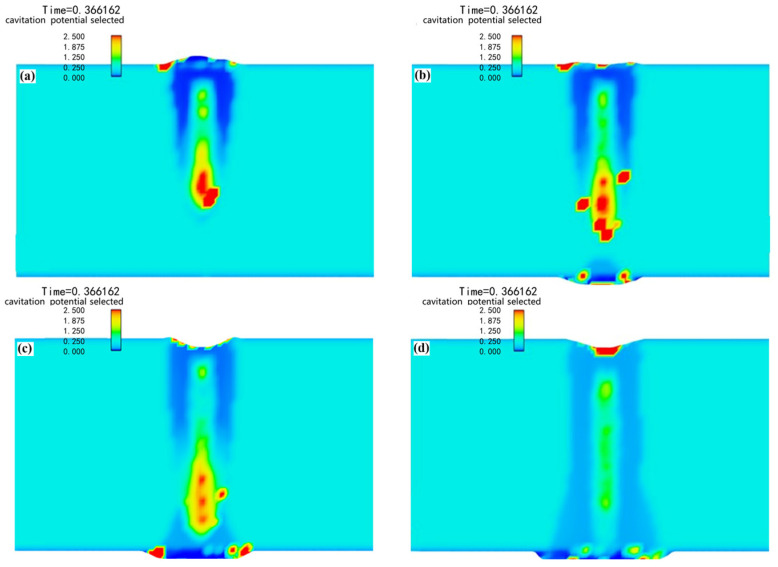
Cavitation potential distribution of 2A12 aluminum alloy welds in different penetration states: (**a**) unpenetrated weld, (**b**) critical penetrated weld, and (**c**,**d**) penetrated weld.

Cavitation bubbles tend to be generated in the molten pool area where the high temperature and the liquid fluctuate sharply [31]. The cavitation potential can be used to measure the possibility of cavitation bubbles in each area of the molten pool. Figure 8 shows that there were areas with high cavitation potential on the surface of both the penetration and the non-penetration weld. However, the cavitation bubbles generated here could smoothly escape from the surface without forming metallurgical pores after the weld was solidified. The cavitation potential value distribution of the joints with different penetration states was significantly different. It was at its highest at the bottom of the weld without penetration. When the joint was critically penetrated, it remained at a high level at the bottom of the weld. However, with further progress of the welding process, the cavitation potential in the penetrated joint was significantly reduced. The cavitation bubbles located at the bottom found it difficult to escape from the molten pool after being generated. Therefore, there was a large number of metallurgical pores distributed at the bottom of the weld, which can be observed on the unpenetrated weld section. The numerical simulations demonstrate good agreement with the experimental observations.

#### 4.2.3. The Influence Mechanism of Beam Current and Penetration State on Process Type Pores

**Figure 9:**
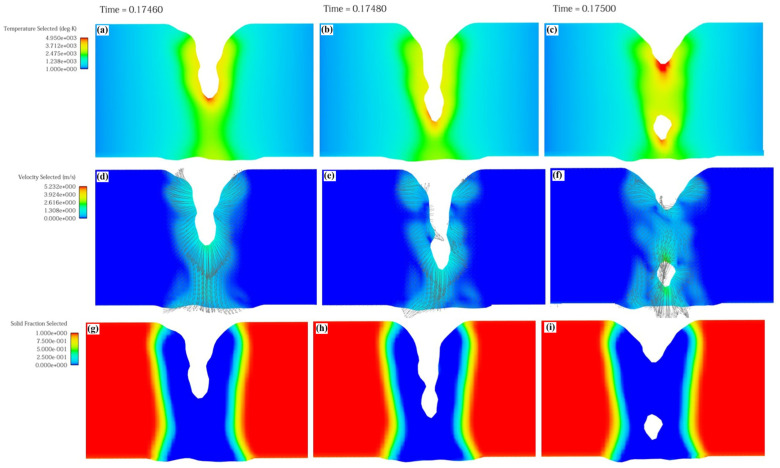
Schematic diagrams of the formation of keyhole bubbles: (**a**–**c**) temperature field, (**d**–**f**) speed field, and (**g**–**i**) molten pool morphology.

Figure 9 shows schematic diagrams of the formation of keyhole bubbles during electron beam welding. During the electron beam welding process, the keyhole will periodically expand and contract, forming a bump on the wall of the keyhole [32]. If the high-power electron beam is irradiated to the raised position, the wall of the keyhole will become straight due to the evaporation of the metal element and the reaction force of the metal vapor, helping the keyhole to maintain an open state. Conversely, if the beam is blocked by the upper bump or the power of the beam reaching the bump is not enough to maintain the keyhole threshold, the keyhole will be closed under the action of surface tension to form bubbles in the liquid metal. Therefore, maintaining a large heat input is beneficial to the stability of the keyhole behavior, and the pore defects in the weld will be reduced as the beam current increases after the joint is penetrated.

The generation of process-type pores is related to the unstable behavior of the keyhole, so the number of bubbles formed by the keyhole collapse can be used to measure the possibility of pore defects. The molten pool state of each parameter entered a quasi-steady state at 6~8 ms, so the number of closed bubbles that formed by the keyhole was counted in the calculation model at the same speed and with different beams, as shown as Table 4. When P = 700 W, the molten pool just penetrated, and after the joint was melted, the number of closed bubbles in the keyhole decreased as the power increased.

**Figure 10:**
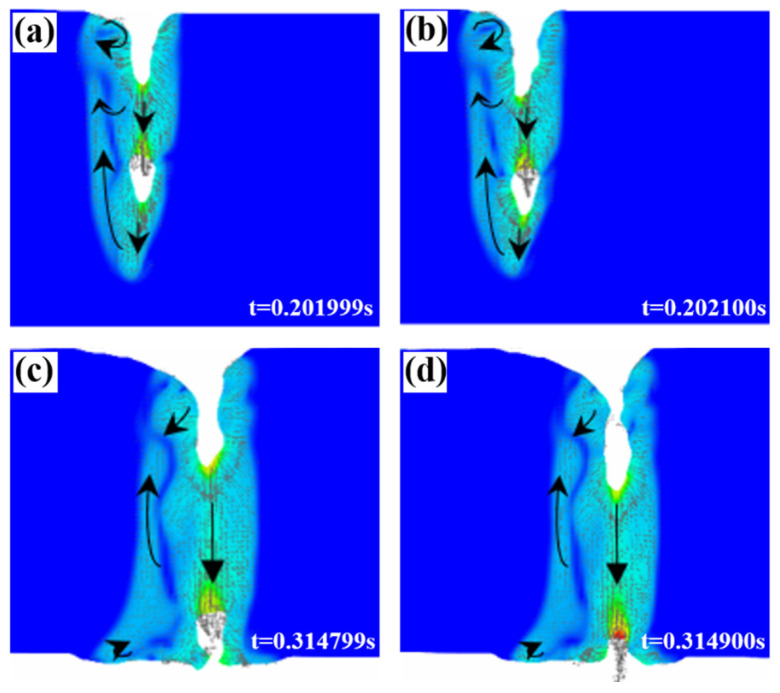
Flow state: (**a**,**b**) unpenetrated welds and (**c**,**d**) penetrated welds.

Figure 10a,b show that in the unpenetrated molten pool, the keyhole cavity will move downward as the depth of the molten pool increases. If the gas cavity cannot be effectively filled with liquid metal due to metal vapor discharge obstruction before reaching the bottom of the molten pool, it will flow backward with the liquid metal at the bottom edge of the molten pool and will not be able to escape in time before the molten pool cools, forming pore defects. Figure 10c,d show the keyhole behavior characteristics of the penetrated welds. Because the bottom of the molten pool provided an outlet, the gas directly escaped from the lower surface after the keyhole cavity reached the bottom of the molten pool, which is conducive to the escape of closed bubbles in the keyhole. Generally speaking, joint penetration is beneficial for the reduction in process-type pore defects. However, the results obtained from the process test showed that the porosity of the joint in the micro-permeable state is the highest, and further analysis is needed.

**Figure 11:**
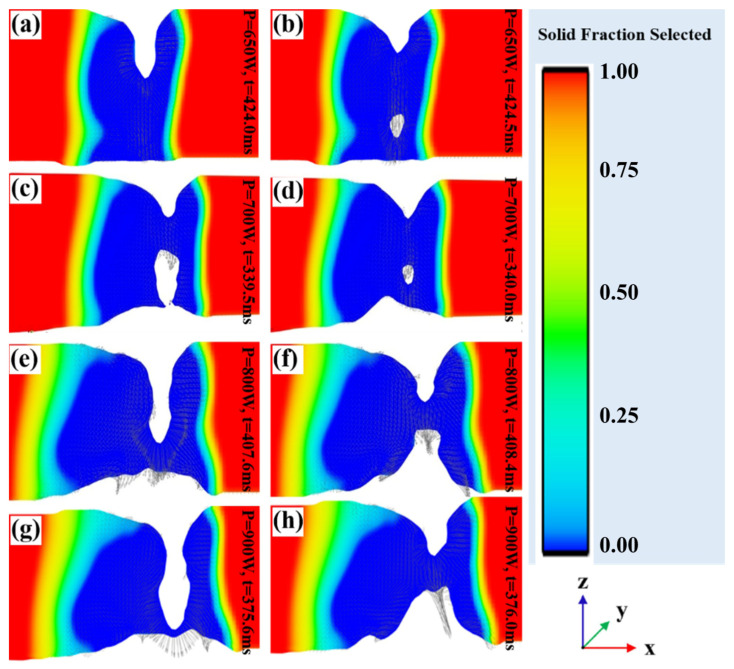
Typical keyhole behavior under different powers at a speed of 1200 mm/min; (**a**) P = 650 W, t = 424.0 ms; (**b**) P = 650 W, t = 424.5 ms; (**c**) P = 700 W, t = 339.5 ms; (**d**) P = 700 W, t = 340.0 ms; (**e**) P = 800 W, t = 407.6 ms; (**f**) P = 800 W, t = 408.4 ms; (**g**) P = 900 W, t = 375.6 ms; and (**h**) P = 900 W, t = 376.0 ms.

Through the analysis, it was found that the energy density of the electron beam reaching the keyhole is the key to maintaining the stability of the keyhole. Therefore, in the vicinity of the critical penetration state of the joint, increasing the welding beam current when the molten pool was not penetrated was beneficial to the stability of the keyhole. As shown in Figure 11a,b, there was a small amount of melting metal on the back of the critically penetrated joint, and the liquid metal at the bottom of the molten pool formed a smaller liquid film, such that greater additional surface tension pressure was generated. When the molten pool behavior reached a quasi-steady state, the metal vapor in the cavity was not able to break through the liquid film and was removed from the lower surface, resulting in no loss of electron beam energy. So, the porosity of the joints was relatively low at the critical penetration state. With continued beam current increase, and when the joint was just penetrated, as shown in Figure 11c,d, a large amount of metal vapor was discharged from the bottom of the molten pool, such that there was not enough metal vapor reaction force to maintain the open state of the keyhole. The keyhole became unstable and formed bubbles. After increasing the beam current again, the metal evaporation effect was enhanced. Even if the upper part of the keyhole closed to form a liquid bridge that can hinder the input of electron beam energy, the metal vapor reaction force inside the keyhole could still discharge the liquid below to form an outlet, as shown in Figure 11e–h. At this time, the closed keyhole cavity will not be formed, so the porosity of the weld is significantly reduced.

#### 4.2.4. The Influence Mechanism of Welding Speed on Pore Defects

**Figure 12:**
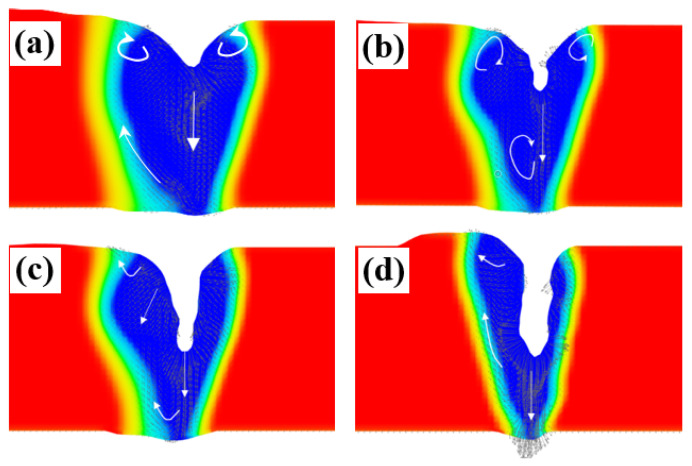
Keyhole and molten pool flow behavior at different welding speeds: (**a**) v = 300 mm/min, P = 500 W; (**b**) v = 750 mm/min, P = 600 W; (**c**) v = 1200 mm/min, P = 700 W; and (**d**) v = 2400 mm/min, P = 900 W.

The keyhole morphology and molten pool flow behavior at different welding speeds are shown in Figure 12. It was found that the higher the speed, the thinner the liquid wall in front of the keyhole, and the more liquid flowed to the back of the molten pool. When v = 300 mm/min and P = 500 W, the depth of the keyhole was stable in a relatively shallow position most of the time, with a lower number of keyhole collapses. The size of the molten pool was large at low speeds, and the convection in the molten pool was stable, thereby reducing the heat loss caused by convection heat transfer [33]. When the welding speed was lower, the molten pool lasted longer, which was conducive to the smooth escape of the generated bubbles, resulting in the low porosity of the weld. Increasing the speed and the power to v = 750 mm/min and P = 600 W, the depth of the keyhole changed within a range. It was found that the radius of the keyhole became smaller, so the keyhole stability became worse more frequently. When the small hole was so unstable that the flow of the electron beam welding pool was turbulent, which was harmful to the escape of bubbles, this affected the generation and floating of the bubbles within the keyhole bubbles [34]. With the further increase in the welding speed and the power, the keyhole radius became larger, the wall surface of the keyhole became more straight, the keyhole behavior became more stable, the liquid flow behind the keyhole became more regular, and there was less eddy current flow inside the molten pool, which were all beneficial for the bubbles’ escape.

## 5. Conclusions

1. When the speed is constant, the porosity of the weld is related to the penetration state. Minimum joint porosity levels were achieved under critical penetration states (0.23% at 300 mm/min; 0.36% at 1200 mm/min), whereas full penetration exhibited an initial increase followed by a decrease in porosity with elevated thermal input. Under critical penetration conditions, the weld porosity first increases and subsequently decreases with rising welding speed, reaching a minimum of 0.23% at 300 mm/min.
2. During electron beam deep penetration welding, the stability of the keyhole depends on the balance between the reaction force of the metal vapor on the wall of the keyhole and the surface tension. Increasing the reaction force of the metal vapor is beneficial for improving the stability of the keyhole. In the unmelted joint, the bubbles can only escape from the upper surface after being generated, so the pore defects are more serious. When the molten pool exhibits micro-penetration, the metal vapor escapes from the lower surface, and the partial loss of the reaction force of the metal vapor reduces the keyhole’s stability. The behavior of the keyhole becomes stable when the beam current continues to increase. The problem of pore defects in the joints depends on two stages of bubble generation and floating. Using small and large specifications during welding is beneficial to stable the keyhole and molten pool flowing behavior, thereby reducing joint pore defects.

## Figures and Tables

**Table 1:** 1060 and 2A12 aluminum alloys (wt%) [23,24].

Component	Cu	Mg	Mn	Fe	Si	Zn	Ti	Al
1060	0.05	0.03	0.03	0.35	0.25	0.05	0.03	99.60
2A12	3.80–4.90	1.20–1.80	0.30–0.90	≤0.50	≤0.50	≤0.30	≤0.15	rest

**Table 2:** Welding process parameters.

Test	Welding Speed *v* (mm/min)	Beam Current *I_b_* (mA)
1	300	25
2	300	26
3	300	27
4	720	31
5	720	32
6	720	33
7	1200	34
8	1200	37
9	1200	41
10	1200	43
11	480	28
12	600	30
13	900	34
14	1500	41

**Table 3:** Distribution and morphology of pores in the longitudinal section at different speeds.

Speed/*v* (mm/min)	Distribution	Binarization
300	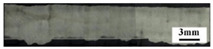	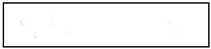
720	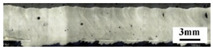	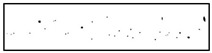
900	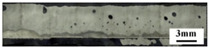	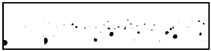
1200	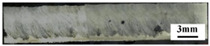	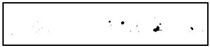
1500	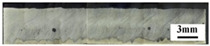	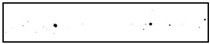

**Table 4:** Numbers of bubbles under different beam currents.

Test	Welding Speed *v* (mm/min)	Power *P* (w)	Numbers of Bubbles
1	1200	600	48
2	1200	650	25
3	1200	700	50
4	1200	800	18
5	1200	850	12
6	1200	900	9

## Data Availability

The original contributions presented in this study are included in the article. Further inquiries can be directed to the corresponding author.
